# Editorial: Rearing, health, and disease management of special economic animals: paving the way for a sustainable special economy through animal agriculture

**DOI:** 10.3389/fvets.2025.1651737

**Published:** 2025-07-28

**Authors:** Izhar Hyder Qazi, Christiana Angel, Graeme B. Martin, Jiping Liu

**Affiliations:** ^1^Guangdong Provincial Key Lab of Agro-Animal Genomics and Molecular Breeding, College of Animal Science, South China Agricultural University, Guangzhou, Guangdong, China; ^2^Shaheed Benazir Bhutto University of Veterinary and Animal Sciences, Sakrand, Pakistan; ^3^Key Laboratory for Agro-Ecological Processes in Subtropical Region, Institute of Subtropical Agriculture, The Chinese Academy of Sciences, Changsha, China; ^4^University of Chinese Academy of Sciences, Beijing, China; ^5^The UWA Institute of Agriculture and UWA School of Agriculture and Environment, The University of Western Australia, Crawley, WA, Australia

**Keywords:** silkworms, honeybee, mink, cashmere goat, ostrich, arctic fox, mulberry, animal agriculture

With the expanding demands of a growing human population, the concept of “Special Economy” has emerged in relation to animal agriculture. In that context, there is attention worldwide on the rearing and utilization of “special economic animals”—the 50 plus species that have been recently “domesticated” for commercial purposes, as a resource for food and fiber. Those developed to provide human food include livestock (e.g., sika deer, red deer, yak) and birds (e.g., silky chickens, pigeons, ostriches). Those developed to provide clothing for humans include fur animals (fox, raccoon dog, mink), and insects (e.g., silkworms). Another insect, the honeybee, provide human food as well as a pollination service (eg, honeybee).

However, the management of these “special economic animals” brings complex pressures and challenges, some of which we have addressed in this Research Topic where we explore a broad variety of topics, including rearing, production, health, and disease. We invited the submission of articles covering theoretical, basic, and applied research on any type of special economic animal and their habitats. We have thus been able to gather diverse insights that contribute to the development of more sustainable practices in this field of high significance to society. Here, we highlight those insights.

## Ruminants of special economic interest

In the Chinese forest musk deer, an endangered species, Xie et al. studied the effect of dietary supplementation of *Epimedium*, a traditional Chinese herb with aphrodisiac and anti-stress properties, on hormone levels, the gut microbiome, and metabolism. The supplement enhanced dry matter intake and feed-gain ratio, as well as the reproductive system. The analysis of the gut microbiota showed that the supplement increased abundance of beneficial bacteria, such as *Firmicutes*, and reduced the abundance of the potentially pathogenic *Proteobacteria*. Metabolomics analysis showed that several key pathways including lipid metabolism, hormone regulation (ovarian steroidogenesis) and antioxidation, were improved. Collectively, these findings indicate that *Epimedium* is beneficial for the rescue of the musk deer, might also be useful for other ruminant livestock.

In the yaks in the Qinghai-Tibet Plateau, the problem is that the transition period, an important determinant of female reproductive efficiency, usually falls in late winter and early spring, when the natural pasture is in very poor supply. Shang et al. therefore studied the effect of perinatal nutritional supplementation and early weaning on serum biochemistry, metabolites, and reproductive efficiency in transitioning yaks. They documented the limitations posed by the conventional grazing system and also report how a nutritional supplement has positive outcomes for glucose, nitrogen, and lipid metabolism, and increases the secretion of reproductive hormones. The outcome is an acceleration of postpartum recovery and therefore improved reproductive efficiency.

With respect to fiber production, the cashmere goat is of special interest. The animals are celebrated for their fine undercoat, produced by the secondary follicles in the skin and harvested to produce high-end textiles that command premium prices in global markets. We present below two papers that provide a molecular biological foundation for improving the yield and quality of cashmere. In the first, Chang et al. identified several key regulatory proteins in secondary follicles. Expression analysis has shown that these proteins were produced more in anagen and less in telogen, particularly in the outer root sheath. Lu et al. used a genome-wide association study to identify 107 candidate genes associated with fleece traits. Additionally, they detected 8 significant single-nucleotide polymorphisms (SNPs) at the genome level and 232 at the chromosome level, with several mutations being related to fleece traits and therefore potentially useful for genetic selection.

## Fur-producing animals of special economic interest

In addition to silk, the fashion industry makes use of high-quality fur produced by the mink and the arctic fox, and the rearing of these two animals has regional economic importance.

In the mink industry, Aleutian disease (mink plasmacytosis), causes spontaneous abortion and death, and thus inflicts huge economic losses. Hu et al. used genotype data to identify genetic markers associated with immune function and resistance to the virus that is responsible. Their work lays a reasonable foundation for a genetic framework based on biological processes that will contribute to improving resilience against the disease. Focussing on mink nutrition, Cao et al. showed that dietary supplementation with the postbiotic, *Enterococcus faecium*, can improve the health and productivity of growing males. They observed accelerated body weight gain, probably explained by improvements in the digestibility of crude protein and dry matter. In addition, *Enterococcus faecium* supplementation enhanced immunity and intestine development.

The Arctic fox exhibits a distinctive lipid metabolism, perhaps due to the extreme environment in which they evolved. The underlying mechanisms are poorly understood, so Zhu et al. used transcriptomics and lipidomics to investigate how the animal handles triacylglycerol and glucose. They found that that 40% dietary crude fat was used for body weight gain, primarily through an increase in accumulation of subcutaneous adipose tissue. Arctic foxes have high levels of triacylglycerol and phosphatidylethanolamine in their adipose tissue, as well as high levels of very-low-density lipoprotein in the liver. By contrast, the levels of free fatty-acids in adipose tissue were normal, so insulin resistance was not a problem. The authors emphasized that the higher fat accumulation capacity and distinct characteristics of hepatic and adipose lipid metabolism, and glucose metabolism, would facilitate glucose homeostasis and enhance fat accumulation.

## Insects of special economic interest

The honeybee is of special interest because it produces honey, and of massive general interest because it is responsible for pollinating food crops that supply up to 90% of the world's human nutrition. It is thus most disturbing that the worldwide honeybee population is under threat from a variety of factors, including diseases and parasites. Mallory et al. reviewed the status of European Foulbrood, a bacterial disease, caused by *Melissococcus plutonius*, that has already caused severe economic damage. They provide an overview on the diversity of causal pathogens, disease distribution, antibiotic resistance, and virulence determinants. They argue that the disease is made more severe by other microbial species that work alongside *M. plutonius*, although they also point out that more evidence is needed. Finally, they present alternative options for combatting the threat of European Foulbrood, hoping to stimulate a discussion on the use of honeybee symbionts and probiotics as therapeutic alternatives.

The silkworm has a long history as an insect of special economic importance and is the subject of three papers. First, focusing on the primary food source of the silkworm, the mulberry, Huang et al. reported on mulberry bacterial blight, a devastating disease that causes serious reduction in the yield and quality of mulberry. Using high-throughput culturomics and metagenomic sequencing technology, they identified 10 causal pathogens. Their study of the distribution patterns of the pathogens and changes in the microbiome community of mulberries revealed *Pseudomonas syringae* and *Pseudomonas fulva* to be most important causal pathogens in the eight provinces of China. Focussing on a serious disease that directly affects the silkworm, pebrine, Qazi et al. carried out an in-depth molecular and phylogenetic characterization of methionine aminopeptidase type 2 (MetAP2) in *Nosema bombycis*, the causative microsporidian parasite. Phylogenetic analysis revealed that the MetAP2 gene and its protein sequence in *Nosema bombycis* were closely related to those of *Nosema* species infecting wild silkworms, but differ significantly from those in other insect microsporidia, fungi, yeasts, and higher organisms, including humans. These findings suggest that conservation of MetAP2 is closely aligned with evolution of the host species, and provide data that may support the improvement and development of diagnostic tools and therapeutic strategies to combat the disease in China's sericulture sector.

The third article on the silkworm, by Wen et al., concerned the potential toxic effects of mercury on growth, development, and antioxidant capacity in the larvae. Following mercury exposure, a histological analysis of both the midgut and fat body of silkworms showed signs of dose-dependent damage. Antioxidant enzyme activity and immune function were also disturbed. Several key genes that control oxidative phosphorylation, nutrient metabolism, hormone biosynthesis, lysosome activity, ribosome biogenesis in eukaryotes, and ribosome pathways, were found to be differentially expressed in the midgut or the fat body of mercury-exposed silkworms. These findings enhance our understanding of the impact of toxic elements on growth and development of the silkworm, and also provide a more general insight into the biological effects of mercury exposure in invertebrate organisms.

## The other species of special economic interest

For the rabbit, Jeong et al. studied a *prion-like protein* gene (*PRND*), a member of the prion protein family, and identified nine novel single-nucleotide polymorphisms (SNPs) that could affect protein function or structure and lead to potential harmful effects. This is preliminary evidence, but it indicates a direction for research into rabbits as partially resistant species.

In the Martina Franca breed of donkey, Fusaro et al. evaluated the effect of dietary supplementation of hemp-based polyunsaturated fatty acids (PUFAs) on the membrane lipid profiles and reproductive performance of males (jacks). The supplement improved sperm morphology, and reduced the peroxidation index and oxidative stress, suggesting improvements in membrane fluidity and oxidative stability, two measures of sperm function.

The only article on birds concerned the ostrich, an animal that provides quality meat, leather, and feathers, as well as other profitable by-products. Chick mortality, often resulting from enteritis, is a serious issue in the ostrich industry, leading to poor outcomes for farmers and breeders. In the search for a nutritional solution, Li et al. supplemented juvenile ostriches with chicory and found that it improved the growth rate and reduced early mortality, perhaps by modulating the intestinal microbiome and mitigating intestinal inflammation.

## Concluding remarks

Rearing, health, and disease management are as important for special economic animals as they are for mainstream livestock. Through proper care, monitoring, and preventive measures, farmers can enhance productivity, animal welfare, and economic returns, and lay a strong foundation for a resilient and sustainable special economy based on animal industries. Although many articles in this collection provide high-quality evidence on various animal species of special economic importance, there remains significant room for further discussion to raise awareness and interest in the rearing of these economically valuable animals.

## Author's note

GM has senior authorship for this article.

